# Luminance gradient at object borders communicates object location to the human oculomotor system

**DOI:** 10.1038/s41598-018-19464-1

**Published:** 2018-01-25

**Authors:** Markku Kilpeläinen, Mark A. Georgeson

**Affiliations:** 1 Department of Psychology and Logopedics, University of Helsinki, Siltavuorenpenger 1A, Helsinki, FI-00014 Finland; 2 School of Life & Health Sciences, Aston University, Birmingham, B4 7ET United Kingdom

## Abstract

The locations of objects in our environment constitute arguably the most important piece of information our visual system must convey to facilitate successful visually guided behaviour. However, the relevant objects are usually not point-like and do not have one unique location attribute. Relatively little is known about how the visual system represents the location of such large objects as visual processing is, both on neural and perceptual level, highly edge dominated. In this study, human observers made saccades to the centres of luminance defined squares (width 4 deg), which appeared at random locations (8 deg eccentricity). The phase structure of the square was manipulated such that the points of maximum luminance gradient at the square’s edges shifted from trial to trial. The average saccade endpoints of all subjects followed those shifts in remarkable quantitative agreement. Further experiments showed that the shifts were caused by the edge manipulations, not by changes in luminance structure near the centre of the square or outside the square. We conclude that the human visual system programs saccades to large luminance defined square objects based on edge locations derived from the points of maximum luminance gradients at the square’s edges.

## Introduction

The visual field location of a given object is arguably the most fundamental object property that our visual system needs to represent and convey. Location information is of obvious importance for avoiding obstacles and other vision-for-action functions. However, object recognition in the human visual system also relies heavily on location information. This is because the processing of virtually all object features and, as a result, our object recognition capabilities deteriorate drastically when the retinal projection of the object lies away from the fovea towards the peripheral retina (for a review, see^1^). For example, faces and digits presented at 10° eccentricity need to be enlarged by a factor of 7 and 3, respectively, in order for identification performance to reach the same level as in foveal viewing^2,3^. Therefore, in order to perform object recognition on the best possible level, it is instrumental for human observers to move the retinal projection of an object of interest to the fovea, i.e., to make a saccadic eye movement towards the object. This, in turn, requires a useful location representation that the oculomotor system can use to program the saccade.

Since most or all of the neural structures that are considered critical for saccade programming are retinotopically organized^4–7^, the location representation of a small, localized saccade target can be assumed to be a relatively simple matter. The topographic location of the neuron(s) activated by the small object indicates the location of the object unambiguously enough. However, the issue becomes more complex when extended objects are considered. Neural responses in the primate early visual system place a great emphasis on the edges of a large object^8–11^. While the central regions of such objects do produce some neural activity, there is no evidence of a central activity peak, which would indicate the location of the object in a straight-forward manner. As a result, one is led to think that the computation of saccades to large objects is based on signals concerning the object’s edges.

It is, in principle, not necessary that a single value for the location of a large object is represented anywhere in the visual system. However, since people viewing naturalistic scenes tend to make saccades *directly to the centres* of large objects, not the edges^12,13^, and since people are easily able to make saccades to the centres of large line drawing shapes^14,15^ such a centre location seems to be computed and communicated to the oculomotor system in an effortless, precise and timely manner.

Here we studied what property of a large object’s edges is used in the computation of a saccade to the object. We asked human observers to make saccades to the centres of large square shapes. The edges of the squares were manipulated from trial to trial such that the two main edge detection theories, one based on the locations of steepest luminance gradient^16,17^, the other based on the locations of maximal contrast energy^18,19^ produce different predictions for the saccade endpoints. Our results clearly indicate that saccades to large luminance-defined objects are programmed based on edge locations derived from the points of steepest luminance gradient.

## Methods

### Subjects

Altogether 11 subjects (6 female, age 22–38) participated in the study. Seven subjects participated in experiment 1, three in each of the other experiments. The subjects who participated in each experiment are indicated in Figures presenting the data. All subjects reported normal visual acuity without optical correction. Subjects S3 and S4 were aware of the purpose of the study, but their data did not differ from the data of other subjects. The study was conducted in accordance with the principles of the Declaration of Helsinki and the guidelines of the University of Helsinki ethical review board, who also approved the study. The participants received a small compensation. The participants signed written informed consent.

### Stimuli and predicted effects of stimulus manipulations

**Figure 1:**
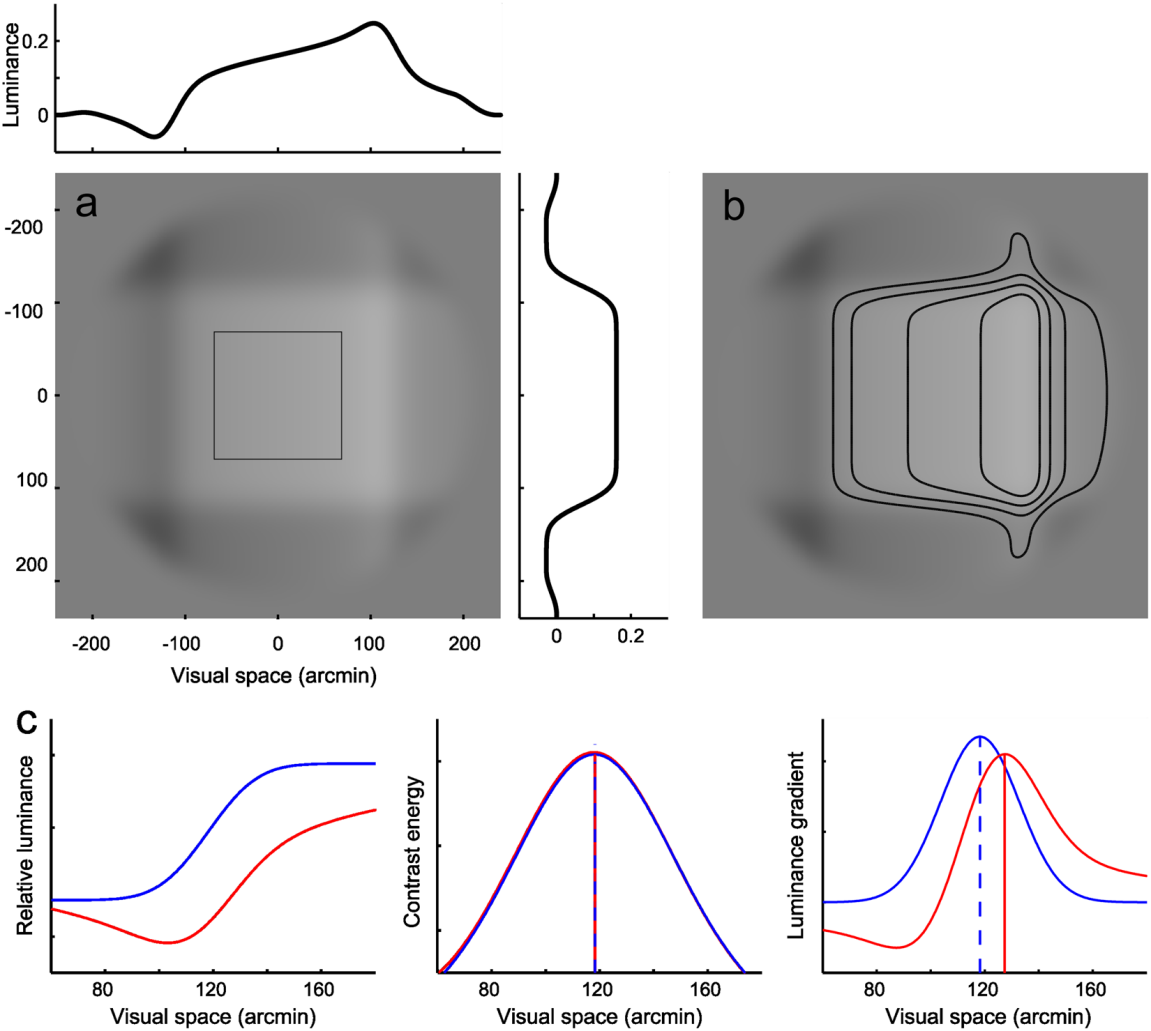
An example of the stimuli used in the study. (**a**) A bright square with horizontal phase +45 degrees and vertical phase 0. Contrast of the images is 1.5 times the real contrast for the purpose of illustration. The dark outline square indicates the size of the hit zone, which was not visible to the subjects. The graphs show the relative luminance cross-sections in the horizontal and vertical direction. (**b**) The same stimulus with iso-luminance contours added. (**c**) Left: Luminance profile of the leading edge of a square with 0 deg phase (blue) and +45 deg phase (red), middle: contrast energy profiles of the two edges, right: Luminance gradient profiles of the two edges.

The stimuli were squares produced by summing two orthogonal 1-D waveforms, such as those defined by Morrone and Burr^19^, see Fig. 1a. Each 1-D waveform was produced by summing sinewaves according to equation 1.

1 $$L(x,\varphi )={\sum }_{h=1,odd}^{99}\tfrac{1}{h}{C}_{0}\,\sin (2\pi {f}_{0}h(x-dx)-\varphi \tfrac{\pi }{180}),$$

where *ϕ* is the phase of the individual harmonics (in degrees), *h* is the harmonic number, *f*_0_ is the fundamental spatial frequency in cycles per waveform (i.e., per 520 pixels (≈7.9°), always 1 in this study), *C*_0_ is the Michelson contrast of the fundamental SF wave (12%), *dx* is a spatial shift of the entire waveform (-period/4, for a bright square, +period/4 for a dark square). The width of the square was thus always about 4° of visual angle.

The stimuli varied from trial to trial in the following manner: the horizontal (left-right) and vertical (up-down) waveforms were manipulated independently by shifting the phase (*ϕ*) of each harmonic of the waveform by a certain amount (−45 to +45 deg, where 360 deg is a full cycle) relative to the harmonic’s wavelength. For example, in Fig. 1a, the phase is 0 in the vertical (y) direction (horizontal edges), and + 45 deg in the horizontal (x) direction (vertical edges). The resulting waveforms were blurred by convolution with a Gaussian filter (SD 16 pixels, 14.6 arcmin if not specified otherwise). The plaid pattern was produced by applying the two waveforms orthogonally to two 520 × 520 pixel squares, and by summing those squares. The plaid was then multiplied by a circular raised cosine window with a 520 pixel diameter, where the outermost 52 pixels (47 arcmin) consisted of the raised cosine edge. The circular window did not affect the rms contrast (12.8%) or the locations of highest luminance gradient or contrast energy in the square.

The stimuli were created with Matlab 8 (Mathworks, Natick, MA, USA), running in a PC with an Nvidia Quadro K5000 (Nvidia, Santa Clara, CA, USA) graphics card, and presented with the Psychophysics Toolbox 3^20^ on a gamma corrected 22.5” VIEWPixx (VPixx Technologies Inc., Quebec, Canada) display with a 120 Hz refresh rate and 16-bit greyscale resolution (in the M16 mode) and background luminance of 104 cd/m^2^. The viewable area of the display subtended 29.2 × 18.5° at a viewing distance of 93 cm.

Figure 1b illustrates how a simple luminance thresholding would predict edge (and square) locations that are clearly incompatible with visual perception. The horizontal edges, for example, do not really appear tilted as the iso-luminance contours would predict. A closer inspection is needed to illustrate the difference between the predictions based on the luminance gradient peaks and contrast energy peaks (which correspond to points of maximum phase coherence in a Fourier transform of the waveform, see^18^). Figure 1c (left) shows how the luminance profile of the leading edge of a square changes when phase is changed from 0 to +45 deg, leaving the contrast energy peak location unchanged (center), thus predicting no change in saccade endpoints. The luminance gradient peak, in contrast, moves by about 9 arcmin (right), predicting a 9 arcmin shift in saccade endpoints.

### Eye tracking

Eye movements were recorded with Eyelink 1000 (SR Research, Missisauga, Canada) video eye tracker at 1000 Hz, using a chin rest. Both pupil and corneal reflection were used for tracking. The eye-tracker was controlled by means of the Eyelink toolbox for Matlab^21^. The calibration targets of the standard 9-point calibration procedure were shifted vertically 10% and horizontally 20% towards the centre from their standard position in order for them to correspond more closely with the stimulus area used in the experiments. In validation, mean error was required to be below 0.4° and maximum error below 0.9°. Calibration and validation were always subject controlled, which tends to yield better calibration accuracy^22^. During the experiments, calibration was repeated if the trial-by-trial drift check error approached 0.5° in a constant general direction or if the subject removed her head from the chin rest, at least twice during each experimental session.

### Procedure

Each trial started with the appearance of a fixation crosshair in the centre of the display, after which the subject indicated stable fixation with a button press. If fixation was stable, a target square appeared after a random delay (600–800 ms) in one of the four possible locations, always at 8° eccentricity, on left or right from fixation and 0.6° above or below the horizontal midline. The subjects were instructed to quickly estimate the location of the centre of the square and move their gaze to that point. Fixation crosshair remained on the screen after the target square appeared, to facilitate voluntary rather than reflexive saccades. If the subject moved their gaze to within the 2.4° wide hit zone (Fig. 1a), which was not visibly marked, they received a feedback (“the saccade was fairly accurate”) and another (“the saccade was somewhat inaccurate”) if the saccade landed elsewhere. During the practice blocks, subjects were instructed that their task was not to maximize the rate of hits, but rather to estimate the centre point of the square and try to reach that the best they could. Nevertheless, subjects’ saccades landed within the hit zone on average 92% (SD over subjects 1.7%) of the time. The hit zone did not move with the phase manipulations, so if it had some effect on the results, it should have decreased the effect of phase manipulations.

In experiment 1, there were 24 blocks of 50 trials (80 trials per condition, 72 for S11). In experiment 2, there were 12 blocks of 54 trials (72 trials per condition). In the control experiments, there were 6 blocks of 48 trials in each (96 trials per condition). Experiment 1 was carried out in two sessions, the other experiments in one session each. There were 2 blocks of 20 practice trials (not included in analyses) at the beginning of each experimental session. In every experiment, each possible combination of the horizontal and vertical phases were presented in the four possible locations an equal number of times, in a balanced and pseudorandomized order.

In natural viewing, where the head is unrestrained, people make gaze shifts with a combination of head and eye movements. However, the gaze shift amplitudes required in this study (8°), which are within a very typical range for free viewing^23^, fall into the range where most people would only move their eyes even if free to move their head^24–26^.

### Data processing

**Figure 2:**
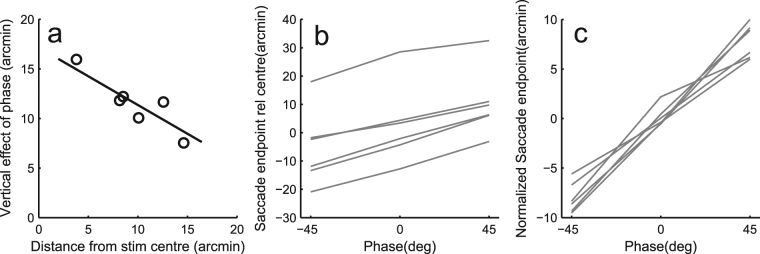
Removing the effect of irrelevant individual saccade tendencies. (**a**) The effect of vertical phase (average saccade endpoint difference between +45 deg and −45 deg conditions) as a function of the vertical undershoot tendency (bias from stimulus centre towards horizontal midline in phase condition 0) for six subjects. R^2 of the linear fit is 0.82. (**b**) Absolute horizontal average saccade endpoint for six subjects. (**c**) To better reveal the effect of phase conditions, data were normalized by shifting the data along the y-axis of the figure so that the mean across phase conditions is 0 for each subject.

Saccades that were clearly outside the square (more than 2.9° from the square centre) were excluded from analyses, leading to an average exclusion rate of 1.1% (max 3%). The excluded saccades were mostly (probably involuntary) saccades with very short (<100 ms) or long (>250 ms) latency and a small (<1°) amplitude. In general, the task seemed to be relatively easy to all subjects. The average saccade endpoints of all subjects were rather close to square centre (max displacement 0.57° horizontal and 0.4° vertical) and saccade endpoint variability, both within subjects (SD range 0.56°–0.72° horizontal, 0.37°–0.46° vertical) and between subjects (SD over subjects 0.31° horizontal, 0.23° vertical) is low relative to the size of the stimulus (about 4°). The stimulus setup was designed so that a constant bias in saccades to one direction (e.g. upwards) or undershoot tendency (hypometricity) of saccades would not significantly hinder the interpretation of results. However, one type of bias could not be *a priori* avoided. There is a tendency for saccades to be somewhat predominantly horizontal^27–29^ and undershoot to large objects is stronger in the vertical direction^13^. Such a bias does not necessarily reflect a special role for the horizontal midline, but could rather be a form of oculomotor range effect (see^30^). Since our stimuli were placed vertically 40 pixels higher or lower than the fixation point, we were able to estimate how much saccades were biased towards the horizontal midline. For each subject and stimulus location, we calculated the saccade endpoints’ mean vertical distance from the horizontal midline. We included only trials with vertical phase 0, where saccades should land 40 pixels above or below the horizon. All subjects showed a bias, where average saccade endpoint distance from the horizon was less than 40 pixels, or 36 pixels (x-axis in Fig. 2a indicates the amount of undershoot). We emphasize that this is not an upward or downward bias, but a bias towards the horizontal midline from both the upper and lower visual fields. Across subjects, there was a strong correlation between the average vertical extent of saccades and the vertical effect caused by the phase shifts. This vertical undershoot tendency seems to compress the vertical extent of all saccades across conditions and, consequently, also compresses the effect of the vertical phase shifts. Thus it seems warranted to adjust the predictions of the vertical effect by the amount of compression caused by the vertical undershoot tendency and we have done so when comparing model predictions with results concerning vertical phase manipulations.

Despite the measures taken to ensure accurate calibration, the absolute average saccade endpoints can never be exactly in the centre of the stimulus. In addition to calibration reasons, this could be due to idiosyncrasies of the saccade system itself^31,32^. These factors should be constant *across phase conditions*. Since the absolute endpoints were not of interest, but rather the differences caused by the phase shifts, we subtracted each subject’s mean of all saccades from the means in different conditions. Figure 2 illustrates the effect of this procedure. Between subject variance (Fig. 2b) is removed by this zero-shifting (Fig. 2c), making the figure more informative, but the effect of phase remains completely unchanged.

### Data availability

The data generated during the current study are available in the Open Science Framework repository, osf.io/b8mn5.

## Results

### Experiment 1: Border signals conveyed by luminance gradients steer saccades to object centre

**Figure 3:**
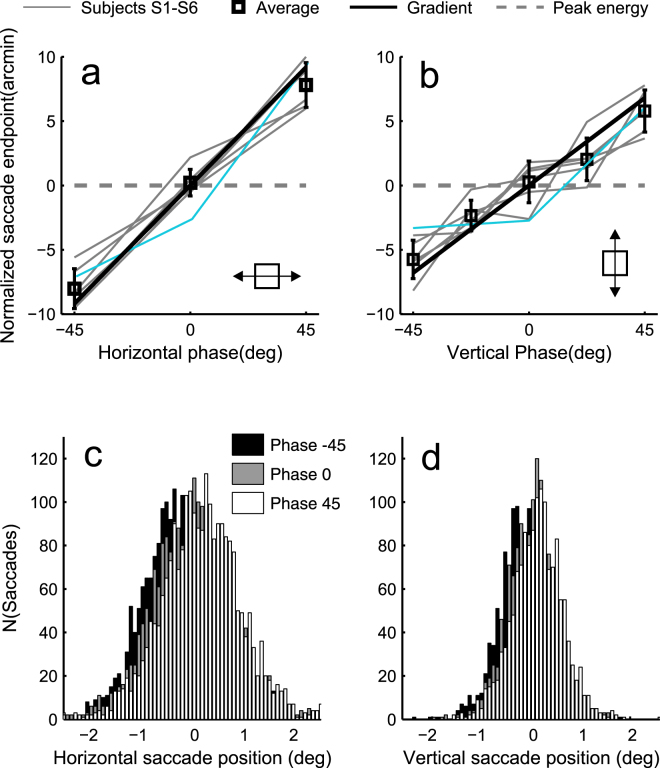
Saccade endpoints closely follow the shifts of luminance gradient peaks at square edges. (**a**) The horizontal position of the saccade endpoint as a function of horizontal phase of the square. (**b**) The vertical position of the saccade endpoint as a function of the vertical phase of the square. Thin grey lines represent individual subjects (S1–S6), the cyan line a subject (S11) for whom stimuli appeared further from the horizontal midline (not included in further analyses), black square markers the means over subjects (errobars ±1 SD). Predictions are based on points of maximum luminance gradient (thick black line) and points of maximum contrast energy (grey dashed line). Insets indicate the direction of square location manipulation. (**c**,**d**) Saccade endpoint distributions of the six subjects in the horizontal and vertical dimension. The sum of all saccades is not the same in (**c**) and (**d**), because in the vertical dimension there were also conditions −22.5 and +22.5 deg, which are omitted from the histogram for clarity.

We manipulated the edges of square objects such that the points of maximum luminance gradients at the *edges* shifted. Saccades aimed at the *centre* of the object shifted in the same direction, in close quantitative agreement. This is illustrated in Fig. 3, where the average saccade endpoints (square symbols) lie very close to the prediction based on luminance gradients (thick black line), with no free parameters, both when the phase manipulation is in the horizontal direction (Fig. 3a) or vertical direction (Fig. 3b). The pattern of results is very similar across subjects (thin grey lines). The data comprehensively contradicts the prediction based on points of maximum contrast energy (dashed thick grey line). To check that the results were not specific to the stimulus locations used, we tested different stimulus locations for one naïve subject (S11). Eccentricity was again 8°, but vertically displaced 5°, rather than 0.6° from the horizontal. His results were very similar to those of other subjects (see cyan line in Fig. 3a,b). His data are not included in further analyses due to a different number of trials and phase conditions.

The effect of phase shifts on average saccade endpoints is not caused by changes in the proportion of some extreme values. This is illustrated by the histograms of all saccades of all subjects in the different phase conditions (Fig. 3c,d). The entire saccade endpoint distribution shifts with the phase shifts.

Mean latencies for different subjects in this experiment were in the range 152 to 237 ms (group mean 193 ms). The possible interaction between phase manipulations and saccade latency was analysed in two ways: Firstly, we conducted a repeated measures ANOVA with each subject’s saccades divided into 5 latency bins with equal number of saccades in each. Secondly we conducted a linear mixed model with Subject as a random variable. In the ANOVA, with vertical saccade endpoint as the dependent variable, the effect of vertical phase was significant (F(4,20) = 43.4, p = 0.001), but the effect of latency bin (F(4,20) = 0.61, p = 0.663) and the phase x latency interaction effect (F(16,80) = 1.29, p = 0.227) were not. The same pattern of results was suggested by a linear mixed model (phase: F(4,7089.042) = 6.09, p = 0.001), latency: F(1,7089) = 0.271, p = 0.603, phase x latency: F(4,7089.058) = 1.568, p = 0.180).

Regarding the horizontal dimension, the ANOVA outcome was similar to the vertical case, i.e., the effect of phase (F(2,10) = 84.6, p = 0.001) was significant, effect of latency bin (F(4,20) = 1.02, p = 0.420) and the interaction effect (F(8,40) = 1.29, p = 0.275) were not. However, here the linear mixed model produced a somewhat different outcome, where all effects were significant (phase: F(2,7093.065) = 11.09, p = 0.001), latency: F(1,7093) = 23.05, p = 0.001, phase x latency: F(2,7093.087) = 3.952, p = 0.019). The effect estimates predict that the effect of phase (phase −45 vs 45 deg) on saccade endpoints increases from 10.5 to 21.1 arcmin when going from the fastest to the slowest saccades. The relationship of latency is unlikely to be strictly linear, but the exact nature of the relationship is beyond the scope of this article.

We conclude that when subjects program a voluntary saccade to the centre of a luminance-defined square object, the computation is based on edge locations derived from the points of steepest luminance gradient.

### Experiment 2: An alternative explanation based on luminance centroid is refuted by the effect of blur size

**Figure 4:**
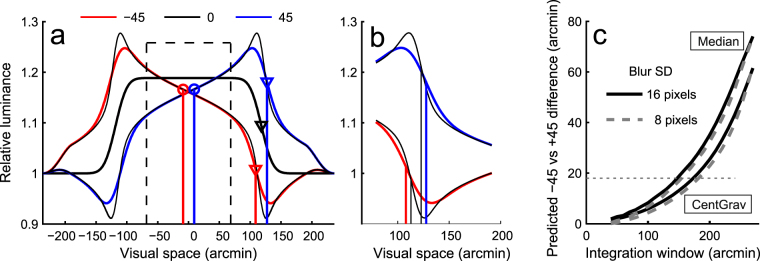
An alternative explanation based on luminance structure near the square centre. (**a**) Luminance cross-sections of a square with phase −45 (thick red line), 0 (thick black line) and 45 (thick blue line). The thin black lines on top of the −45 and 45 curves correspond to the same phase conditions, but with blur halved. The vertical lines from the curves indicate predicted saccade endpoints, based on points of maximum luminance gradients at edges (triangles) and luminance centroids (circles). (**b**) A close-up of the trailing edges of the −45 and 45 waveforms in (**a**) and the saccade endpoint predictions of the peak luminance gradient model. Line colours as in (**a**). With a smaller blur level, the predicted saccade endpoints are closer to each other (black vertical lines), i.e., effect of phase is smaller. (**c**) The prediction by the luminance centroid operator strongly depends on the size of the integration window and is essentially the same for 16 and 8 pixel blur regardless of exact method of calculation (see labels). The 8 pixel prediction has been shifted down by 1 pixel to reveal the two curves. The dashed horizontal line indicates the difference predicted by peak luminance gradient at edges.

When the edges of the square are manipulated by changing the phase of the harmonics, the luminance structure in the central area of the square also changes (see luminance waveforms in Fig. 4a). It would, in principle, be possible that the centre of the square is determined by means of some sort of luminance centroid computation. Figure 4a illustrates the main idea behind the centroid schemes we considered. Luminance is integrated from within a certain central window (dashed rectangle). When phase is non-zero, the calculated luminance centroid moves somewhat towards the side with the peak luminance (red vs. blue circle). The magnitude of change predicted by this scheme strongly depends on the size of the integration window (Fig. 4c). Most predictions are completely unrealistic, but with a small subset of integration window sizes this framework predicts changes in saccade endpoints that are in agreement with the data (and the predictions based on luminance gradients at the edges, see red vs. blue triangles in Fig. 4a and the dashed horizontal line in Fig. 4c). However, whereas the luminance gradient model predicts the effects of phase to decrease with smaller blur (thick vs thin vertical lines in Fig. 4b), the centroid scheme predicts virtually identical results in different blur conditions (solid vs. dashed curves in Fig. 4c). Experiment 2 tested these two different predictions by presenting squares with 8 pixel blur SD, rather than 16. Three subjects (S4–S6) participated in this experiment. To avoid a confounding learning effect, the order of participating in experiments 1 and 2 was balanced across the three observers.

The luminance centroid was determined numerically from the central horizontal cross-section of an oversampled (4×) version of the stimulus: One way to calculate the centroid (label CentGrav in Fig. 4c) is given by the centre-of-gravity calculation, Equation 2.

2 $$\tfrac{1}{S}{\sum }_{x=-\tfrac{1}{2}w}^{\tfrac{1}{2}w}L(x)x,$$

where *x* is pixel location, *S* is ${\sum }_{x=-\tfrac{1}{2}w}^{\tfrac{1}{2}w}L(x)$, *L* is luminance of a pixel and *w* is the width of the integration window. However, since it is not clear how the visual system should treat strong intensities near the edges of the integration window, a less outlier-sensitive median of the luminance distribution (the point which divides the luminance mass within the integration window to two equal halves, label Median in Fig. 4c) was also considered.

**Figure 5:**
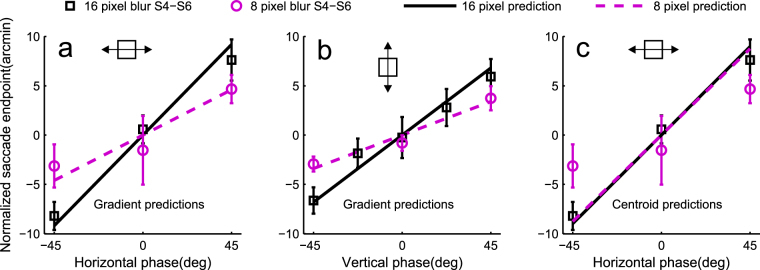
The edge luminance gradient theory predicts the effects of blur size, the centroid theory does not. (**a**,**b**) Data and predictions derived from the luminance gradient model, for 16 pixel blur (black squares and solid black line) and for 8 pixel blur (magenta circles and dashed magenta line). (**c**) Data are the same as in (**a**), but predictions are derived from the luminance centroid calculations. Errobars ±1 SD.

The results are quite clear. When blur size was smaller, the effect of phase changes was smaller, for all subjects and for both horizontal and vertical directions. The change is captured exquisitely by the model based on edge luminance gradients (Fig. 5a,b), again with no free parameters. While the luminance centroid model (with a handpicked integration window size) can predict one set of data quite well (for example the data for the 16 pixel blur condition if Fig. 5c), it then fits the other (8 pixel blur) data set poorly, as the prediction is essentially the same for both conditions (Fig. 5c). In addition to arguing strongly against the luminance centroid explanation, the result provides additional support for the gradient peak model: it is not only a better predictor of the effect of blur, but a rather good one. The predictions of the gradient peak model are in good agreement with the data for both levels of blur (Fig. 5a,b). In particular, halving the blur reduces the effect of phase (45 vs −45 deg) by 49 and 53% in the horizontal and vertical directions, respectively, while the predicted reduction was 50%. To quantify the performance difference between the two models, we calculated Akaike’s AIC for the two model fits (see ^33^). The SSE (sum of squared errors), K (number of parameters +1) and N (the number of data points) for the gradient model are 7.3, 1 and 12, respectively and 30.8, 2 and 12 for the centroid model. The models were fitted by least squares to the averaged data. The data points that were only measured for the 16 pixel blur condition (±22.5 deg) were not included. The AIC analysis indicated that the gradient model was more than 10^4^ times more likely to be the better model for the data.

### Control experiments: Results are the same without the ‘ears’ and with ‘real’ stimulus offsets

**Figure 6:**
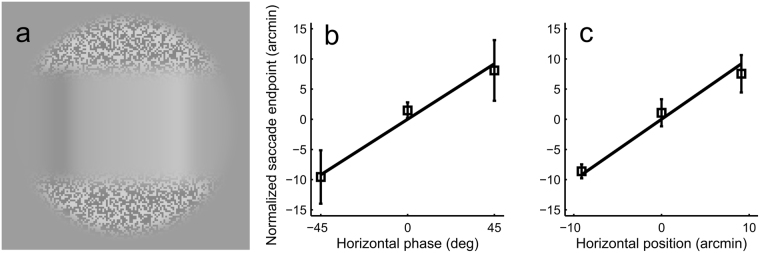
The results of control experiments. (**a**) To control for the possible effect of the ‘ears’ outside the corners of the square (see Fig. 1a), a horizontal waveform identical to that used in experiment 1 was embedded into binary noise. In this example the square has +45 deg horizontal phase, but no ‘ears’ appear outside the corners of the square. (**b**) The data with such a stimulus still followed the luminance gradient prediction. Subjects S7–S9. (**c**) When the square was moved by actually offsetting it within the circular window (keeping phase always 0), average saccade endpoints changed in an appropriate manner. Subjects S8–S10. Errobars ±1 SD.

The circular window was used in this study to extract a square-like stimulus out of a periodical plaid pattern. An unavoidable side effect of the windowing was the dark or light ‘ears’ outside the corners of the square (Fig. 1a). The contrast of the ears changed quite considerably with phase changes. This could have affected saccade direction even in the absence of any effect of the square edges. To rule out such a confounding effect, we ran a control experiment in which there was a horizontal waveform identical to that in the earlier experiments, bounded above and below by binary noise (see Fig. 6a). The noise had the same mean luminance as the horizontal waveform, but double the contrast. The vertical transition from the waveform to the noise followed the same function as the luminance transition in a waveform with phase 0 in the earlier experiments (see black thick line in Fig. 4a). Also, we had so far assumed that a phase change that moves the luminance gradient peak by 10 pixels (9.1 arcmin) should be expected to result in 10 pixel change in the saccade endpoint and that this amount of change should not be affected by the location of the circular window, display shape etc. We tested the validity of this assumption with the stimulus in Fig. 6a, but by actually moving the square (phase always 0) within the circular window horizontally by ±10 pixels. Three subjects participated in each control experiment, all of whom had not participated in the earlier experiments, in order to preclude the unlikely confound that subjects would simply repeat the pattern of eye movements they had learned in earlier experiments. The results of the control experiments were the same as in experiment 1. Saccade endpoints changed appropriately with stimulus manipulations (Fig. 6b,c). The mean effect of phase/stimulus shifts on horizontal saccade endpoints was 17.7 arcmin (SD 9.4) in the ‘ear’ control experiment, 16.2 acrmin (SD 4.1) in the ‘real shift’ experiment and 15.8 arcmin (SD 3.1) in experiment 1.

## Discussion

In the current study, human observers were instructed to make saccades to the *centre* of a square stimulus, while the locations of the squares’ *edges* were varied from trial to trial. The average saccade endpoints were very close to the square centre, yet followed the edge shifts in remarkable agreement. Our results point to a simple and efficient principle for the guidance of saccades to the centres of extended objects, a task that the human visual system frequently and successfully performs. For objects with luminance-defined edges, the points of steepest luminance gradient at the edges of the object are sufficient for computing a useful estimate of the object location.

The importance of saccadic eye movements for normal human visual behaviour cannot be overemphasized. A defining limitation of our visual system is the drastic decrease in our visual acuity when moving from central to peripheral visual field^34–36^. In addition to neural undersampling^37^, this loss of acuity is also due to crowding and attentional effects^38,39^. The eye movement system is usually studied with very small localized stimuli. While such a paradigm has revealed many interesting properties of the eye movement system, it lacks some ecological validity, as in more naturalistic conditions people frequently need to bring rather large objects to foveal processing (and attentional focus). Nuthmann and Henderson^13^ showed that people do so by making a saccade directly to the central region of such objects (see also^40^). This ability is not a special case of naturalistic stimuli as people can make saccades to the centres of line drawings as easily as to smaller, point-like stimuli^14,15^. Outside the laboratory, saccades are guided, along with low-level stimulus properties, by subject’s intentions and objects’ higher level attributes^41,42^. These factors should rarely completely preclude the effect of object’s edge locations. Indeed, Linden, Mathôt & Vitu^43^ found that first saccades are predominantly drawn to the physical centre of an object even with objects that have a strong motor priming potential (see also^44^), although task might have a more dominant effect when a motor plan concerning an actual physical object is already ongoing *before* the saccade to the object is initiated (cf.^45^). Further work is needed to chart the extents to which the saccade guidance principle observed here applies to various stimuli with varying properties (e.g., shapes, sizes, eccentricities and border types). However, the gradient peak approach has been shown to predict the locations where people report edges and bars across a wide range of waveforms, contrasts and blurs^17,46,47^, so a fair amount of generalizability is to be expected.

The visual system places a strong emphasis on discontinuities, such as the boundaries between different luminance levels. Many visual illusions, such as the Craik-O’Brien - Cornsweet illusion and simultaneous contrast illusion suggest that the perceived brightness of an object is strongly influenced by the contrast at the border between the object and its background and the physical luminance of a uniform area around the object centre is unavailable, as such, to conscious perception^48,49^. Such perceptual level emphasis on edges is mirrored by neural responses in the primate visual system^8–11^, where responses to luminance boundaries are generally stronger than responses to more central regions of the stimulus. In the primate visual cortex, this edge dominance of neural responses is often particularly pronounced in the initial phase of the responses. Then, in tens or hundreds of milliseconds, the activity spreads towards the centre of the figure representation^11,50,51^. Although the time-courses of the filling-in at neural and perceptual levels^52,^^53^ are, in some cases, in agreement, the matter is still unresolved. In the current study, shorter saccade latency attenuated the effects of edge manipulations, but to a rather limited degree. Only horizontal edge manipulations were significantly affected, and even the fastest saccades were still affected by phase manipulations. It is possible that the latency modulation is due to the fastest saccades being less carefully aimed at the square centre, and more reflexively directed towards the entire stimulus pattern in general (see Fig. 1a). Nevertheless, it is also possible that latency does modulate the effects of phase manipulations, perhaps because of incomplete filling-in. This could be even more strongly the case if subjects were pressed to produce quick saccades, which was not the case in this study. However, the range of average saccade latencies across subjects was 152–237 ms, which is quite typical, and not longer than fixations during natural viewing^54^. Considering that sensory information ceases to affect saccade programming some tens of milliseconds before saccade execution^55–58^, it seems that at least the slower, object-centre-driven forms of figure-ground-modulation (see eg.^53^) were not needed in our task. We also studied the possible effect of the square’s central area by presenting stimuli with two levels of blur, which moves the points of maximum luminance gradient, but leaves central luminance structure largely unaffected. When blur level was decreased by 50%, the effect of phase shifts decreased roughly by 50%, as predicted by the luminance gradient theory. In addition to offering further support for the maximum luminance gradient theory, this result argues strongly against a decisive role of the central luminance structure of the stimulus.

It has been quite thoroughly established that edges are a fundamental piece of information for the visual system. The next question must then be: what are edges? Edges are salient local changes in one or more properties, such as luminance, colour or texture. This work has only considered luminance-defined edges. Although other cues are often present and definitely relevant^59,60^, luminance is undoubtedly one of the most salient edge cues. Finding edges in a visual scene is generally considered an essential (first) step for useful artificial image analysis systems. By far the most prevalent method for this is the gradient based method^16^, but a contrast energy based method has also gained popularity^18^. Evidence exists for the relevance of both approaches in human visual processing^17,19,61^. The current study unequivocally supports the luminance gradient based approach. Out of the 16 datasets produced by the 11 subjects and 4 experiments, there were none where the saccade endpoints failed to follow the predictions of the luminance gradient account. Although we were here most directly interested in how the central or overall location of a large object targeted by saccades is affected by edge signal manipulations, the current study does provide an indirect measure of the perceived location of edges. Rather than making any conscious judgment of the edges, the subjects’ task was simply to move their eyes to the centre of the square. In fact, edges were not even mentioned in the instruction to the subjects. It could be argued, however, that despite its indirect manner, the current method of measuring edge location judgements is more relevant for natural visual behaviour than the more direct methods of previous studies: people use the information of object edges much more often for searching (voluntarily or reflexively) for the next fixation target than consciously inspecting object boundaries.

Georgeson, May, Freeman and Hesse^47^ have presented a comprehensive computational model which predicts many aspects of human edge perception, including perceived locations of edges. Although they made no strong claims about the underlying physiological wiring, they pointed out that their architecture of two sequential filter-rectify steps with the receptive field size smaller in the first than the second step resembles the sequence of simple and complex cells of the primary visual cortex^62,63^. Apart from indicating a relatively fast neural system, the current results cannot reveal the neural origin of the edge signals the subjects used in directing their eye movements. The results do, however, provide quite precise, testable predictions for the edge signals that should be present on at least one level of the neural system that transforms the vast light pattern landing on retina to the unambiguous, solitary location that the saccades rather densely accumulate around.
